# Nile Tilapia (*Oreochromis niloticus*) *Patched1* Mutations Disrupt Cardiovascular Development and Vascular Integrity through Smoothened Signaling

**DOI:** 10.3390/ijms25063321

**Published:** 2024-03-15

**Authors:** Xiang Liu, Changle Zhao, Lei Liu, Xi Peng, Jianeng Li, Wenjing Tao, Deshou Wang, Jing Wei

**Affiliations:** 1 Integrative Science Center of Germplasm Creation in Western China (CHONGQING) Science City, Key Laboratory of Freshwater Fish Reproduction and Development (Ministry of Education), Laboratory of Aquatic Science of Chongqing, School of Life Sciences, Southwest University, Chongqing 400715, China; liuxiang1@email.swu.edu.cn (X.L.); changle0@email.swu.edu.cn (C.Z.); ll87v5@email.swu.edu.cn (L.L.); nights@email.swu.edu.cn (J.L.); taoeva@swu.edu.cn (W.T.); 2 Sichuan Industrial Institute of Antibiotics, School of Pharmacy, Chengdu University, No. 2025, Chengluo Avenue, Chengdu 610106, China; pengxi@cdu.edu.cn

**Keywords:** Nile tilapia, Hedgehog signaling pathway, Patched1, cardiovascular development, blood circulation, vascular integrity

## Abstract

Hedgehog (Hh) signaling is crucial in cardiovascular development and maintenance. However, the biological role of Patched1 (Ptch1), an inhibitory receptor of the Hh signaling pathway, remains elusive. In this study, a Ptch1 ortholog was characterized in Nile tilapia (*Oreochromis niloticus*), and its function was investigated through CRISPR/Cas9 gene knockout. When one-cell embryos were injected with CRISPR/Cas9 targeting *ptch1*, the mutation efficiency exceeded 70%. During 0–3 days post fertilization (dpf), no significant differences were observed between the *ptch1* mutant group and the control group; at 4 dpf (0 day after hatching), about 10% of the larvae showed an angiogenesis defect and absence of blood flow; from 5 dpf, most larvae exhibited an elongated heart, large pericardial cavity, and blood leakage and coagulation, ultimately dying during the 6–8 dpf period due to the lack of blood circulation. Consistently, multiple differentially expressed genes related to angiogenesis, blood coagulation, and heart development were enriched in the *ptch1* mutants. Furthermore, Smoothened (Smo) antagonist (cyclopamine) treatment of the *ptch1* mutants greatly rescued the cardiovascular disorders. Collectively, our study suggests that Ptch1 is required for cardiovascular development and vascular integrity via Smo signaling, and excessive Hh signaling is detrimental to cardiovascular development.

## 1. Introduction

Hedgehog (Hh) signaling plays a crucial role in embryonic development and multiple tissue homeostasis [1,2]. It is highly conserved across different species [3,4,5]. In vertebrate, there are three types of Hh ligands, namely sonic Hh (Shh), desert Hh, and Indian Hh. The Hh ligands can bind to either of the two inhibitory receptors (Patched (Ptch) 1 and Ptch2) for signaling transduction through the Hh canonical pathway, which is mediated by de-repression of the transmembrane protein Smoothened (Smo) and subsequently activates downstream transcription factors—glioma-associated oncogenes (Gli), or through the Hh noncanonical pathway independent of Smo and/or Gli [6,7].

Numerous studies have shown that Hh signaling plays a critical role in heart development and angiogenesis [8,9,10,11]. In mice, the absence of *Smo* in cardiomyocytes can lead to cardiac hypertrophy, ventricular dilation, atrioventricular canal defects, and myocardial tissue fibrosis [12,13]. Regarding angiogenesis, suppression of Hh signaling through mutations of Hh signaling components such as Shh, Smo, and Gli can cause dysplasia of coronary artery [14,15,16], aortic defect and microvascular sparsity [17,18,19], abnormal capillary permeability [20], and cerebrovascular leakage [21]. Meanwhile, overactivation of Hh signaling through treatment with Shh protein or Smo agonists and deletion of Ptch1 can lead to abnormal dilation of the dorsal aorta and promotes tumor angiogenesis [22,23,24,25], implying that the Hh signaling has angiogenesis-promoting activity. Controversial evidence exists that does not support the angiogenesis-promoting activity of Hh signaling. For example, Ptch1 deficiency or Hh ectopic expression leads to decreased vascular density in embryos [26,27,28]; *Ptch1* overexpression leads to vascular endothelial growth factor (VEGF)-related pathological angiogenesis in the retina [29]; and zebrafish with double mutations of *ptch1* and *ptch2* have defects in the formation of cardinal veins and the establishment of blood circulation [30]. Meanwhile, excessive vascular sprouting leads to increased development of superficial vessels in the eyes of *Smo*-deficient zebrafish [31]. Therefore, to date, the role and potential mechanisms of Hh signaling pathways in cardiovascular biology remain elusive. The biological role of the inhibitory receptor of Hh signaling Ptch1 and its downstream members in heart development and vascular development need to be further investigated.

Nile tilapia (*Oreochromis niloticus*) is an important commercial farmed fish which belongs to the family Cichlidae in the order Cichliformes [32]. It is a good model for developmental biology because of its copious spawning, transparent larval stage, and feasible gene editing, among other qualities [33,34]. In this study, Ptch1 from Nile tilapia was characterized, and then its role in cardiovascular development and vascular integrity during embryonic development and early larval stage was comprehensively investigated. Through CRISPR/Cas9 gene knockout and antagonist treatment, our data indicate that *ptch1* deficiency (i.e., excessive Hh signaling) can disrupt the cardiovascular development and vascular integrity through Smo signaling.

## 2. Results

### 2.1. cDNA Cloning and Sequence Analyses

**Figure 1:**
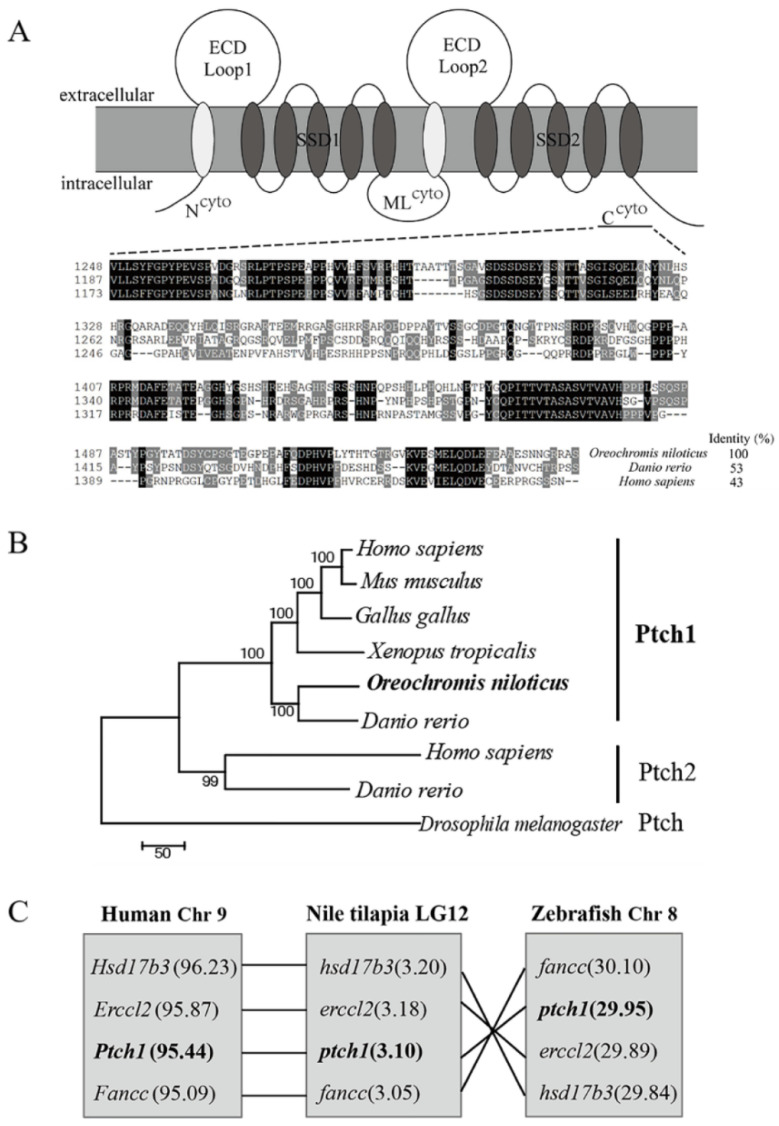
Sequence analyses of Ptch1 from Nile tilapia. (**A**) Putative domains and the amino acid sequence alignment of C^cyto^. (**B**) Phylogenetic tree. The tree was constructed using the neighbor-joining method within the MEGA7.0. The length of the line is proportional to the evolutionary distance of the species from the branching point. Node values represent percent bootstrap confidence derived from 2000 replicates. *Homo sapiens*, PTCH1, NP 000255.2, PTCH2, NP 003729.3; *Mus musculus*, Ptch1, NP 032983.1; *Gallus gallus*, Ptch1, NP 990291.3; *Xenopus tropicalis*, Ptch1, XP 031746817.1; *Oreochromis niloticus*, Ptch1, XP 013127348.1; *Danio rerio*, Ptch1, NP 001292471.1, Ptch2, NP 571063.2; *Drosophila melanogaster*, Ptch, NP 523661.2. (**C**) Syntenic analysis. Ensembl Genome Browser (Ensembl 108 version) was used for analysis. Nile tilapia *ptch1* is located on chromosome LG12, zebrafish *ptch1* is located on chromosome 8 (Chr 8), and human *Ptch1* is located on chromosome 9 (Chr 9). ECD-Loop, extracellular loop domain; SSD, sterol-sensing domain; N^cyto^, cytoplasmic domain of N-terminal; ML^cyto^, cytoplasmic domain of middle loop; C^cyto^, cytoplasmic domain of C-terminal.

The full open reading frame (ORF) of Nile tilapia *ptch1* is 4656 nucleotides in length, located in the genome chromosome LG12 with 49676 bp and 24 exons. It encodes putative 1551 amino acids (Figure S1). Ptch1 is a twelve-pass transmembrane protein and contains three cytoplasmic domains (N^cyto^, ML^cyto^, C^cyto^), two extracellular domains (ECDs) (Loop1, Loop2), and two five-pass transmembrane sterol-sensing domains (SSDs) (SSD1, SSD2). The alignment of the Ptch1 amino acid sequences of Nile tilapia, zebrafish, and human shows high sequence identity between ECDs and SSDs, up to 90% (Table 1). However, the cytoplasmic domains (especially the C^cyto^ domain) exhibit significant variability, with the amino acid identity of the Ptch1 C^cyto^ domain in Nile tilapia being only 50% for zebrafish and 40% for humans (Figure 1A). Phylogenetic analysis shows that vertebrate Ptch1 and Ptch2 are clustered into two distinct subclades with *Drosophila* Ptch as an outgroup, and Nile tilapia Ptch1 follows the evolutionary relationship in vertebrates (Figure 1B). Moreover, the genome location of genes including *hsd17b3*, *erccl2*, and *fancc* adjacent to *ptch1* displays high collinearity between mammals and fish (Figure 1B). Collectively, these data support that Nile tilapia Ptch1 is a true ortholog of mammal Ptch1 (Figure 1C).

### 2.2. Ptch1 Is Required for the Survival of Early Larvae of Nile Tilapia

The *ptch1* mRNA was ubiquitously expressed throughout embryonic and early larval development stages and adult tissues (Figures S2 and S3). In the early larvae at 6 days post fertilization (dpf), high expression of *ptch1* mRNA was found in the heart, brain, and eyes through in situ hybridization (ISH) detection (Figure S2B).

**Figure 2:**
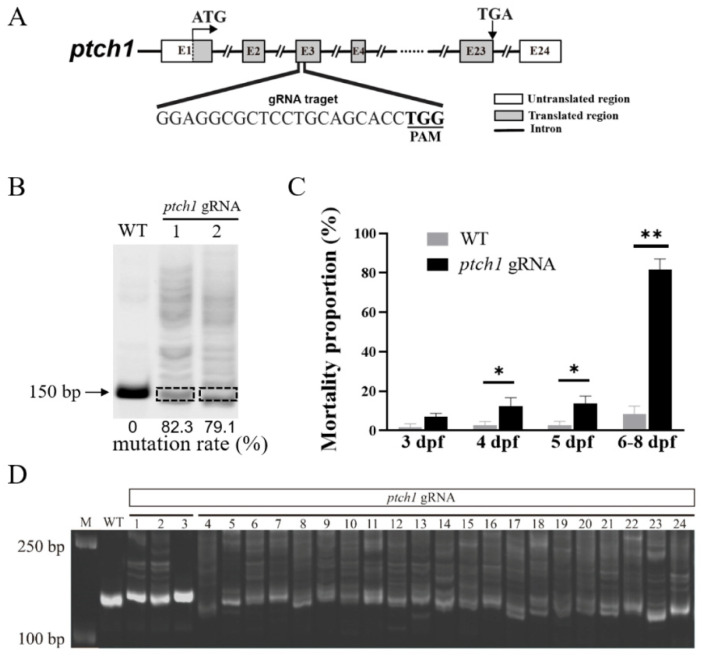
Nile tilapia *ptch1* mutants by CRISPR/Cas9. (**A**) Design of *ptch1* gRNA targeting exon 3. (**B**) Assessment of editing efficiency of *ptch1* gRNA at 2 dpf. Following microinjection of *ptch1* gRNA and Cas9 mRNAs into one-cell embryos, the mutations of the target site were detected by PAGE. The *ptch1* mutation rate for each embryo was calculated as the ratio of the gray value of the mutant bands to that of the total bands. In lanes 1–2, each sample contains one embryo. Arrow indicates the original band of WT *ptch1*. (**C**) Mortality statistics. After the normal development stage (2 dpf), the percentages of dead embryos in *ptch1* knockout embryos (*n* = 294) and WT embryos (*n* = 227) at different developmental stages (3–8 dpf) were calculated. Significant differences versus the control are indicated by * *p* < 0.05 and ** *p* < 0.01. (**D**) Mutation detection of *ptch1* mutants by PAGE at 6 dpf. The mutation rate of 24 abnormally dead embryos in *ptch1* mutant group was detected. Lanes 1–3, embryos with low mutation rate (lane 1 and 2) or with no mutation (lane 3). Lanes 4–24, embryos with high mutation rate. Each sample contains one embryo. PAM, protospacer adjacent motif; M, DNA Marker; WT, wild-type Nile tilapia embryos without *ptch1* gRNA and Cas9 mRNAs co-injection; *ptch1* gRNA, the embryos co-injected with *ptch1* gRNA and Cas9 mRNAs.

To investigate the role of *ptch1*, it was knocked out by CRISPR/Cas9. Briefly, embryonic animal pole was injected with the combinations of *ptch1* gRNA (500 ng/μL) and Cas9 mRNAs (1000 ng/μL) at the one-cell stage. Control embryos were injected only with the same concentration of *ptch1* gRNA or Cas9 mRNAs, and the injection of external RNAs alone did not affect the normal development of embryos. The mutation efficiency of the *ptch1* mutant group was detected at 2 dpf, and the mutation rate of single embryos was up to 79% (Figure 2A,B). During 0–3 dpf, no significant differences were observed between the *ptch1* mutant group and the control group (Figure S4); starting from 4 dpf (0 day after hatching), the mortality rate of the *ptch1* mutant group gradually increased, reaching 80% at 8 dpf, while the mortality rate of the control group was less than 10% (Figure 2C). Further mutation detection indicated that almost 90% of the dead larvae at 6 dpf in the *ptch1* mutant group showed a high mutation rate (Figure 2D). These results suggest that Ptch1 is required for the survival of early larvae of Nile tilapia.

### 2.3. ptch1 Mutations Cause Pericardial Edema and Abnormal Cardiac Morphology

**Figure 3:**
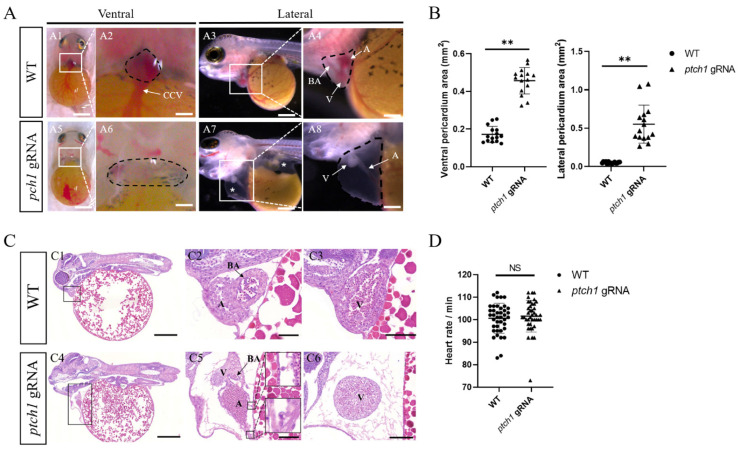
*ptch1* mutations caused pericardial edema and abnormal cardiac morphology in early larvae at 5 dpf. (**A**) Pericardial edema and abnormal cardiac morphology in *ptch1* mutants. The ventral views (**A1**,**A2**,**A5**,**A6**) and lateral views (**A3**,**A4**,**A7**,**A8**) of WT and *ptch1* mutant larvae are shown. The magnified images of heart in (**A1**,**A3**,**A5**,**A7**) are displayed as (**A2**,**A4**,**A6**,**A8**), with black dashed circles indicating the pericardial cavity. Asterisks indicate pericardial edema and dorsal vitelline edema. (**B**) Area of pericardial cavity. The pericardial area in ventral view and lateral view in WT and *ptch1* mutant larvae were calculated using Image J (version 1.54i) software at the same magnification (*n* = 15). (**C**) H&E staining of heart. The pericardial cavity of *ptch1* mutants (black frame in (**C4**)) was larger than that of WT (black frame in (**C1**)). In WT, blood cells were evenly distributed in the heart, and atrium and ventricle were closely connected to the surrounding body wall (**C2**,**C3**). In *ptch1* mutants, there was a significant accumulation of blood cells in the atrium, and the atrial and ventricular wall was significantly separated from the adjacent body wall (**C5**,**C6**), where apoptotic cells were observed (black frame in (**C5**)). (**D**) Statistics of heart rate. The number of heart beats per minute was recorded in the WT group (*n* = 40) and *ptch1* mutant group (*n* = 40). CCV, common cardinal vein; BA, Bulbus arteriosus; A, atrium; V, ventricle. Significant differences versus the control are indicated by ** *p* < 0.01. NS indicates no significant difference. Scale bars, (**A1**,**A5**), 1 mm; (**A3**,**A7**,**C1**,**C4**), 500 μm; (**A2**,**A4**,**A6**,**A8**,**C2**,**C3**,**C5**,**C6**), 100 μm.

To further investigate the cause of death of early larvae of the *ptch1* mutants, heart development of the wild-type (WT) and *ptch1* mutant larvae was observed and compared during 5–6 dpf, i.e., the heart differentiation stage of Nile tilapia [34]. In the WT, heart showed a prominent curvature accompanied by the expansion of the pericardial cavity, marking the division between the atrium and ventricle (Figure 3(A3)). The atrium in the triangular-like pericardial cavity (in the lateral view) was connected to the common cardinal vein (CCV), and the ventricle was connected to the bulbus arteriosus (BA) (Figure 3(A1,A4)). In the heart, blood from the CCV flowed through the atrium, ventricle, and BA and was pumped out into the branchial arch aorta for new circulation (Video S1). By contrast, in the *ptch1* mutant larvae, the heart showed obvious pericardial edema, and the pericardial area was significantly larger than that of WT in the ventral and lateral views (Figure 3A,B). Notably, in addition to pericardial edema, *ptch1* mutants also appeared to have dorsal vitelline edema (asterisks in Figure 3(A7)). In the abnormally enlarged pericardial cavity of the *ptch1* mutants, the stretched and linear atrium was observed, which connected the ventricle and CCV, and there was no blood flowing through the heart (Figure 3(A6,A8) and Video S2).

Hematoxylin and eosin (H&E) staining further revealed abnormalities in the morphology and structure of the heart and pericardial cavity in the *ptch1* mutants. First, the space between the atrium/ventricle and the body wall of the *ptch1* mutants was larger than that of WT, corresponding to the edema of the pericardial cavity in the mutants (Figure 3(C1,C4)). Second, the stretched atrium was accompanied by a thin atrial wall and a large accumulation of blood cells (Figure 3(C5)). Furthermore, some apoptotic cells were observed around the pericardium, which had the characteristics of round cells and chromatin fragments, while it was not observed in WT (Figure 3(C5)). In addition, the abnormal accumulation of blood cells in the atrium of the mutant also indicated that the heart function was affected, although the average heart rate of the *ptch1* mutants was not different from that of the WT at 5 dpf (Figure 3D and Videos S1 and S2). Based on these results of cardiac structure, we speculate that the stretched atrial morphology and apoptosis of pericardial or myocardial cells might be responsible for the abnormal enlargement/edema of the pericardial cavity. Collectively, these results suggest that Ptch1 is critical for cardiac development and function.

### 2.4. ptch1 Mutations Impair Angiogenesis and Vascular Integrity

To investigate the effect of *ptch1* mutations on angiogenesis and vascular integrity, the status of the vascular network and blood flow of WT and *ptch1* mutant larvae were observed and compared at 4–6 dpf, i.e., the period of vascular establishment and development in Nile tilapia [34].

In the WT early larvae at 4 dpf (the time of blood circulation initiation), the blood from the heart tube (the immature heart) circulates through the aortic arch and then circulates in the head, yolk, and trunk–tail regions, and ultimately returns to the heart tube. In the control group, all early larvae developed normally (proportion of normal larvae to total larvae: 82/82), and no larvae with defects of the cardiovascular system were found (Figure S5(A1–F1) and Video S3). In the mutant early larvae at 4 dpf, about 10% (8/65) of the *ptch1* mutants showed defective vessel formation in the whole body (Figure S5(A2–F2) and Video S4), and these mutants (termed ‘*ptch1* mutant Ⅰ’ in this study) with vascular network defects and no blood flow could not survive at 4–5 dpf.

**Figure 4:**
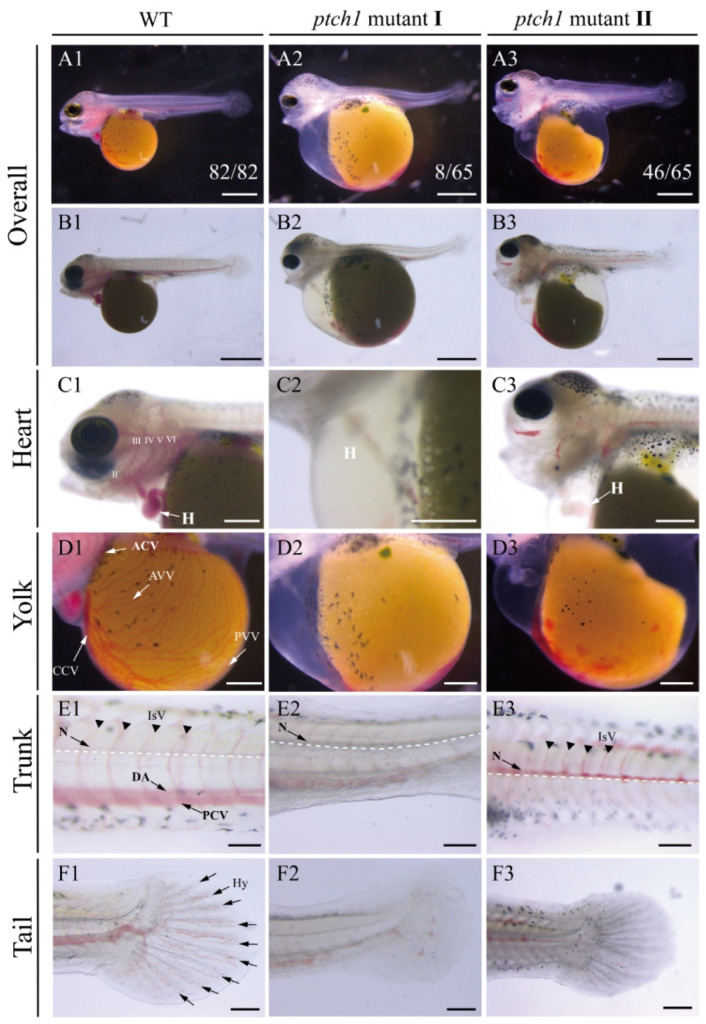
*ptch1* mutations impaired angiogenesis and blood circulation in early larvae at 5 dpf. The numbers in (**A1**–**A3**) represent the proportion of early larvae with this phenotype to the total number of early larvae in the control or mutant group at 5 dpf, respectively. Compared with the WT larva (**A1**–**F1**), the formation of blood vessels of *ptch1* mutant Ⅰ was impaired (**A2**–**F2**). *ptch1* mutant Ⅱ had the phenotype of blood leakage and coagulation (**A3**–**F3**), although they had formed some blood vessels earlier. Images of vascular distribution in tissues such as heart (**C1**–**C3**), yolk (**D1**–**D3**), trunk (**E1**–**E3**) and tail (**F1**–**F3**) are shown, respectively. H, heart; Ⅱ, aortic arch 2 (AA2) in the hyoid arch; Ⅲ, AA3 in the first branchial arch; Ⅳ, AA4 in the second branchial arch; Ⅴ, AA5 in the third branchial arch; Ⅵ, AA6 in the fourth branchial arch; ACV, anterior cardinal vein; AVV, anterior vitelline vein; PVV, posterior vitelline vein; CCV, common cardinal vein; N, notochord; IsV, intersegmental blood vessel; DA, dorsal aorta; PCV, profundal caudal vein; Hy, hypural region. Scale bars, (**A1**–**B3**), 500 μm; (**C1**–**C3**), 250 μm; (**D1**–**D3**), 200 μm; (**E1**–**E3**), 50 μm; (**F1**–**F3**), 100 μm.

The period of branchial arch differentiation, intersegmental blood vessel (IsV) formation, and capillary network branching in Nile tilapia occurs at 5 dpf. During this period, the vascular network was further developed, and WT early larvae had obvious aortic arches in the branchial arch and more vascular branches in the yolk sac and trunk (Figure 4(A1–F1) and Videos S5 and S6). In contrast to the WT, about 71% (46/65) of *ptch1* mutants (termed ‘*ptch1* mutant II’ in this study) exhibited defective aortic arch, yolk sac vascular network (including anterior vitelline vein (AVV) and posterior vitelline vein (PVV)) and IsV, resulting in a defect in vascular network integrity and loss of blood flow (Figure 4(A3–F3) and Videos S7 and S8). In addition, blood leakage and coagulation of multiple tissues, including branchial arch, yolk sac, trunk, and tail, were observed, and the *ptch1* mutant II was not viable at 6–8 dpf (Figure 4(A3–F3) and Figure S5(A4–F4)). Nevertheless, in the mutant group, the average heart rate of *ptch1* mutants (*ptch1* mutant I and *ptch1* mutant II) at 5 dpf did not differ from that of the WT, and the mutants without cardiovascular phenotype had normal heart morphology, vascular network integrity, and heart rate similar to the WT larvae (Figure 3D).

**Figure 5:**
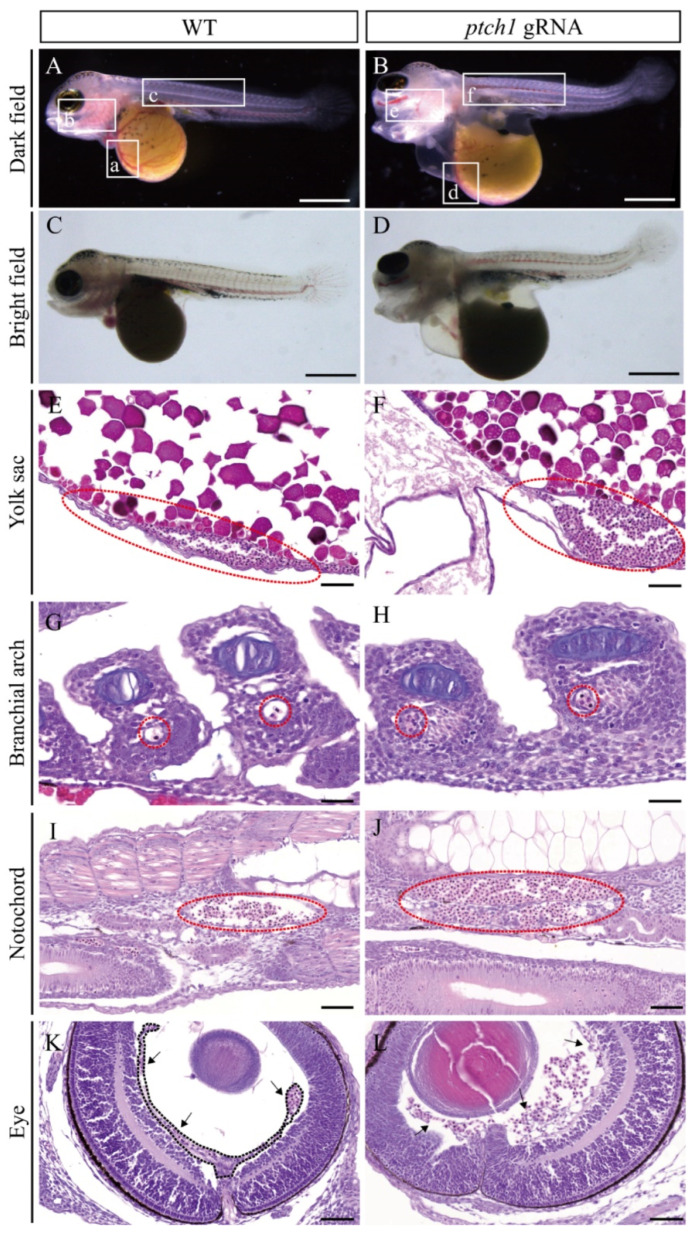
*ptch1* mutations caused lumen dilatation and vascular rupture. (**A**–**D**) Dark field (**A**,**B**) and bright field (**C**,**D**) images of WT and *ptch1* mutant larvae at 6 dpf. Compared with normal blood circulation of WT larvae (frames a, b, and c in (**A**)), the *ptch1* mutants showed blood leakage and obstructed blood circulation (frames d, e, and f in (**B**)). (**E**–**L**) H&E staining. The blood vessels in the ventral yolk sac (**E**,**F**), branchial arch (**G**,**H**), and near the notochord (**I**,**J**) were markedly dilated, and there were numerous blood cells accumulated in the vascular lumen (red dashed circle). Compared with the intact retinal blood vessel in WT (arrows in (**K**)), the retinal blood vessel of *ptch1* mutants was ruptured (arrows in (**L**)). Scale bars, (**A**–**D**), 500 μm; (**E**–**L**), 50 μm.

H&E staining showed that the *ptch1* mutants had significant vascular dilation and blood cell accumulation in the vascular lumens, such as the cardinal vein of the yolk sac, aortic arch of the branchial arch, and IsV near the notochord (Figure 5E–J). Moreover, there were ruptured retinal blood vessels in the *ptch1* mutants (Figure 5I), which might be the reason for the deformed eye morphology of the *ptch1* mutants (Figure S6). By contrast, no abnormalities were observed in the angiogenesis and vascular integrity in the control group (Figure 5A–K).

Taken together, these data suggest that Ptch1 is critical for angiogenesis and vascular integrity.

### 2.5. The Differential Expression Profiles of the ptch1 Mutant and WT Larvae

**Figure 6:**
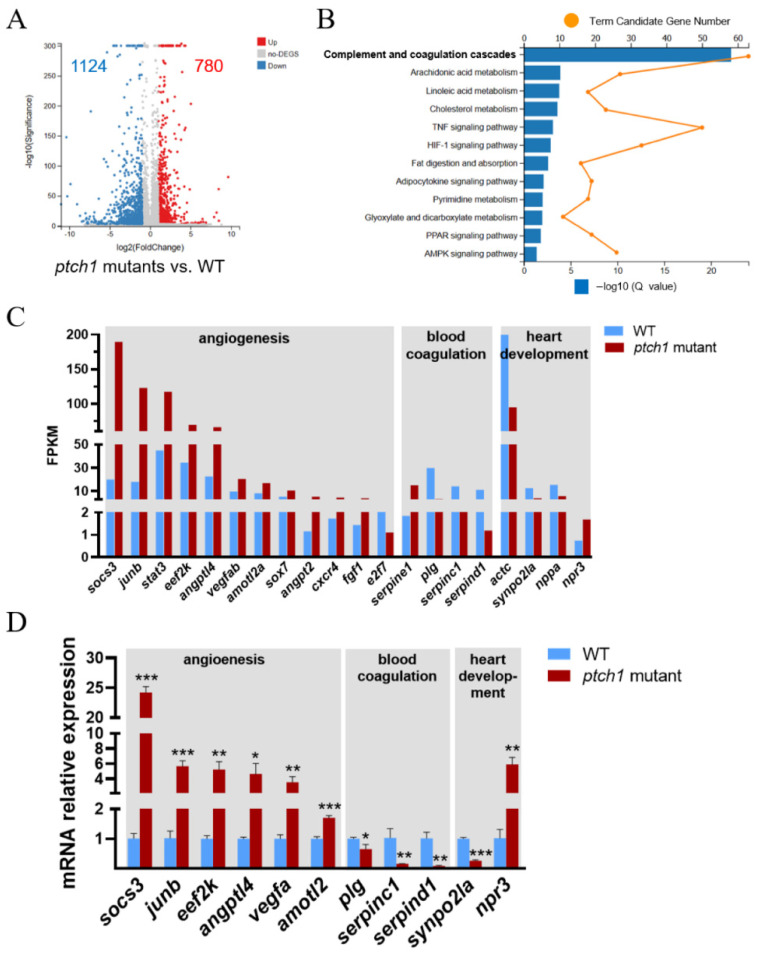
Transcriptome data analyses of *ptch1* mutants and verification by RT-qPCR. (**A**) Volcano plot of differentially expressed genes (DEGs) from WT and *ptch1* mutant early larvae. There were 780 up-regulated genes (red dots) and 1124 down-regulated genes (blue dots) in the *ptch1* mutants. (**B**) KEGG enrichment analysis of DEGs. (**C**) The FPKM values of selected genes in the *ptch1* mutants and WT. Transcriptomic data showed altered expression levels of genes related to angiogenesis, blood coagulation and heart development following *ptch1* mutation. FPKM, Fragments Per Kilobase of exon model per Million mapped fragments. (**D**) RT-qPCR confirmation of selected genes in the *ptch1* mutants. Three biological replicates were conducted, and significant differences were determined using Student’s *t*-test (*n* = 3). The values are represented as mean ± SE (error bars). Significant differences versus the control are indicated by * *p* < 0.05, ** *p* < 0.01, and *** *p* < 0.001.

RNA sequencing analysis was performed to assess the differential expression profiles between the *ptch1* mutant and WT larvae at 5 dpf. A total of 1904 differentially expressed genes (DEGs) were identified (Figure 6A). Through KEGG enrichment analysis, 1124 of DEGs were downregulated in the *ptch1* mutants and mainly enriched in pathways of complement and coagulation cascades and lipid metabolism (Figure S7A), whilst 780 of DEGs were upregulated and enriched in several inflammatory pathways, such as the Janus kinase/signal transducer and activator of transcription (JAK/STAT) signaling pathway, tumor necrosis factor (TNF) signaling pathway, and hypoxia-inducible factor 1 (HIF-1) signaling pathway (Figure 6B and Figure S7B). Further analysis indicated that the genes related to physiological or pathological angiogenesis, such as suppressor of cytokine signaling 3 (*socs3*), JunB proto-oncogene, AP-1 transcription factor subunit b (*junb*), signal transducer and activator of transcription 3 (*stat3*), eukaryotic elongation factor 2 kinase (*eef2k*), angiopoietin-like 4 (*angptl4*), and vascular endothelial growth factor A (*vegfa*), were upregulated, whilst the genes related to anticoagulation, such as plasminogen (*plg*), serpin peptidase inhibitor, clade C, member 1 (*serpinc1*), and serpin peptidase inhibitor, clade D, member 1 (*serpind1*), were downregulated (Figure 6C). In addition, there were several DEGs associated with myocardial proteins (actin alpha cardiac muscle (*actc*) and synaptopodin 2-like a (*synpo2la*)) and blood pressure regulation (natriuretic peptide A (*nppa*) and natriuretic peptide receptor 3 (*npr3*)), suggesting that the affected heart function of the mutant was associated with abnormal cardiac morphology (Figure 6C). These DEGs were further confirmed by RT-qPCR (Figure 6D). Collectively, our data suggest that the DEGs are involved in angiogenesis, and blood coagulation, and heart development, which might be attributed to *ptch1* mutations.

### 2.6. The Rescue of ptch1 Mutants by Smo Antagonist Treatment

**Figure 7:**
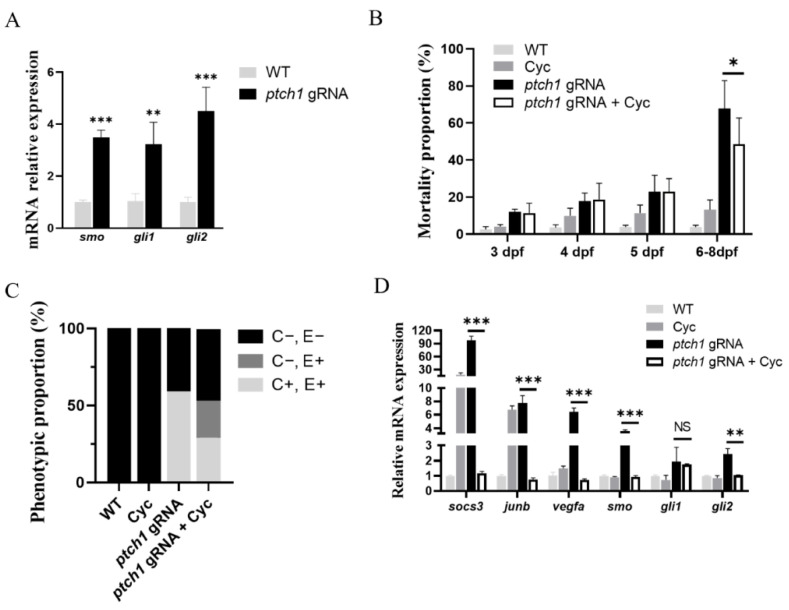
Smo antagonist treatment rescued abnormal phenotypes and mRNA expression levels in *ptch1* mutants. (**A**) mRNA expression of Hh pathway members in *ptch1* mutants by RT-qPCR. (**B**) Mortality in the cyclopamine treatment group and the mutant group. (**C**) The proportion of three types of phenotypes in *ptch1* mutants after cyclopamine treatment at 6 dpf. C−, E−: larvae with normal blood circulation and pericardial cavity, and no pericardial and dorsal vitelline edema. C−, E+: larvae with normal blood circulation and pericardial cavity, but had dorsal vitelline edema. C+, E+: larvae with blood leakage and coagulation, pericardial edema, and dorsal vitelline edema. (**D**) Cyclopamine treatment rescued mRNA expression of the related genes in the *ptch1* mutants. WT, wild-type Nile tilapia embryos; Cyc, WT embryos treated with 10 μM cyclopamine; *ptch1* gRNA, embryos co-injected with *ptch1* gRNA and Cas9 mRNAs; *ptch1* gRNA + Cyc, *ptch1* mutant embryos treated with 10 μM cyclopamine. Three biological replicates were conducted and significant difference was determined using Student’s *t*-test (*n* = 3). The values are represented as the mean ± SE (error bars). Significant differences versus the control are indicated by * *p* < 0.05, ** *p* < 0.01, and *** *p* < 0.001. NS indicates no significant difference.

The mRNA expression levels of Hh canonical pathway members including *smo*, *gli1*, and *gli2* were upregulated in the *ptch1* mutants (Figure 7A). To further investigate whether Ptch1 mediates the cardiovascular development and vascular integrity via Smo, the *ptch1* mutants were treated with Smo antagonist (10 μM cyclopamine) or the same volume of DMSO as the control. At 8 dpf, the mortality rate of *ptch1* mutants treated with cyclopamine was about 48%, whereas the control was over 60% (Figure 7B), suggesting that cyclopamine treatment can decrease mortality caused by *ptch1* mutations.

Based on the status of blood coagulation and edema in *ptch1* mutants, three phenotypes were artificially classified to evaluate the rescue effect by cyclopamine treatment (Figure S8). First, the larvae termed as “Coagulation−, Edema− (C−, E−)” were viable with normal circulatory system (including DA, PCV, and IsV) and normal pericardial cavity, which were similar to the WT (Figure S8A,D,G). Second, the larvae termed as “Coagulation+, Edema+ (C+, E+)” were not viable at 6–8 dpf because of blood leakage and coagulation, pericardial edema, and dorsal vitelline edema (Figure S8B,E,H), which resembles the *ptch1* mutant Ⅱ. Third, the larvae defined as “Coagulation−, Edema+ (C−, E+)” had normal blood circulation, with dorsal vitelline edema but no edema in the pericardial cavity (Figure S8C,F,I). The C−, E+ larvae were viable for the subsequent developmental stages, even though they had dorsal vitelline edema that is a characteristic of high *ptch1* mutation rate. The proportions of *ptch1* mutants with high mutation rate (the C+, E+ and C−, E+ larvae) were similar in both *ptch1* mutant group (59.05%) and cyclopamine treatment group (54.1%) (Table 2 and Figure 7C). Differently, in the cyclopamine treatment group, the proportion of viable larvae (the C−, E− and C−, E+ larvae) with normal blood circulation improved to 70.9% compared to 40.95% in the *ptch1* mutant group (Table 2 and Figure 7C). Furthermore, the expressions of genes related to angiogenesis and Hh downstream (except for *gli1*) in cyclopamine-treated C−, E+ mutants were downregulated to the level of WT (Figure 7D). These results indicate that cyclopamine treatment can alleviate the edema phenotype and rescue blood circulation in *ptch1* mutants, implying that Ptch1 might mediate the cardiovascular development and vascular integrity via Smo signaling.

## 3. Discussion

Although the important role of Hh signaling in cardiovascular development has been extensively demonstrated, there is still controversy over whether the activation of Hh signaling promotes or inhibits cardiovascular development. Moreover, due to the complexity of the Hh pathway and its multiple interactions with other pathways, its underlying mechanisms in cardiovascular development are still elusive. In this study, our work indicates that *ptch1* mutation in Nile tilapia leads to cardiac dysplasia, angiogenesis defects, and vascular leakage through CRISPR/Cas9 gene knockout. As an inhibitory receptor of the Hh pathway, *ptch1* mutations mean the constitutive activation of the Hh signaling. Hence, our study suggests that overactivation of Hh signaling can disrupt cardiovascular development rather than promote it. Meanwhile, the cardiovascular disorders in *ptch1* mutations were greatly rescued by Smo antagonist treatment. Therefore, combining existing research and our findings, we propose the view that excessive or defective Hh signaling is harmful to cardiovascular development, and only appropriate Hh signaling is necessary for cardiovascular development.

To date, our knowledge about the inhibitory receptors of Hh pathway in cardiovascular development remains elusive. In mouse, *Ptch1* deletion leads to death, with an underdeveloped heart, defect of the capillary branching network in cephalic plexus, and dilation of the dorsal aorta during embryo vascular patterning [25,35]. In zebrafish, double mutations of *ptch1* and *ptch2* lead to defective migration of angioblast and failure in circulatory establishment, whereas both *ptch1* and *ptch2* mutants have normal blood circulation [30]. In this study, the role of Ptch1 in the cardiovascular development and vascular integrity of Nile tilapia was comprehensively investigated. At 4–6 dpf, the *ptch1* mutants had abnormal atrioventricular structure, pericardial edema, and defective formation of the blood vessel network in multiple tissues, including the vascular network in the yolk sac and the intersegmental vascular network in somites (Figure 3, Figure 4 and Figure S5). In addition, the *ptch1* mutants exhibited vascular rupture and blood leakage in the retina, yolk sac, and trunk of *ptch1* mutants from 5 dpf onwards (Figure 5), when vascular branching occurs. Our data support that Ptch1 is essential for heart development, vascular network formation, and vascular integrity. These results are consistent with a study of capillary network defects in *Ptch1*-depleted mice reported by Coultas et al. [25], but different from the study of impaired blood circulation in zebrafish with double mutation of *ptch1* and *ptch2* [30]. Therefore, our study suggests that the constitutive activation of Hh signaling caused by *ptch1* mutations leads to cardiovascular developmental disorders rather than promoting angiogenesis.

The cardiac phenotype of *ptch1* mutant larva at 5 dpf was characterized by a stretched atrium, enlarged pericardial cavity (pericardial edema), and edema of the dorsal yolk sac where immature organs such as spleen, kidneys, and intestines were located (Figure 3(A8)). Multiple factors such as cardiac pumping insufficiency, chamber myocardial injury and chamber looping defect, alterations of ion channels and osmotic imbalance of body fluids may contribute to the phenotype of pericardial and yolk sac edema. In zebrafish, reports have shown abnormalities in the development and function of the heart or arteries, such as cardiac pumping insufficiency with atrial blood cell accumulation [36] and developmental defects of the aortic arch [37], leading to pericardial edema. There is a phenotype of elongated heart and cavity ring defects in zebrafish heartstrings (*hst*) mutants, where the T-box domain encoding the Tbx5 transcription factor is located in the *hst* gene [38]. Tbx5 can participate in atrioventricular development and cardiomyocyte proliferation and apoptosis by promoting the transcription of target genes such as calcium/calmodulin-dependent protein kinase ll beta 2 (*camk2b2*), N-Myc downstream-regulated gene 4 (*ndrg4*), NK2 homeobox 5 (*nkx2.5*), etc. [39,40]. The zebrafish *tbx5* mutant has an elongated heart, linear atrium, and pericardial edema, which might be attributed to abnormal cardiac differentiation and myocardial cell growth [39]. In this study, the *ptch1* mutants at 4 dpf showed defective blood vessel formation and signs of heart insufficiency, such as absence of blood flow (Figure S5(A2–F2)). At 5 dpf, the *ptch1* mutants (*ptch1* mutant I and II) showed ischemic ventricle, congestion of blood in the atrium, and pericardial edema, implying that cardiac function might be affected, although the average heart rate of the *ptch1* mutants was not different from that of the WT (Figure 3). Therefore, we speculate that the heart insufficiency and defects in angiogenesis might cause pericardial edema in *ptch1* mutants at 5 dpf. Like *tbx5* mutants, the *ptch1* mutants also displayed a stretched and liner heart, indicating that differentiation of the atrium and ventricle might be affected. This viewpoint is supported by our transcriptome analysis results, such as myocardial related DEGs (*synpol2a*, *npr3*, *nppa*), and the H&E staining results, such as apoptotic cells observations in the pericardium or myocardium of the *ptch1* mutants at 5 dpf (Figure 3C and Figure 6D).

Alterations of ion channels and osmotic imbalance of body fluids may also cause phenotypes of pericardial and yolk sac edema. These have been reported in the drug-treated (e.g., triphenyl phosphate) or gene-knockdown zebrafish, such as the *wwox* mutants with high calcium levels and fluid osmosis imbalances in the heart tissue and putative digestive organs [41], and the *sept7b* mutants with glomerular filtration barrier defects and renal fluid accumulation [42]. In this study, 9 DEGs encoding potassium ion, calcium ion, and gated channel proteins were identified in the *ptch1* mutants, including potassium inwardly rectifying channel subfamily J member 1a (*kcnj1a*), potassium voltage-gated channel, Isk-related family member 4 (*kcne4*), transient receptor potential cation channel subfamily C member 6a (*trpc6a*), and others [43,44,45,46], implying that alterations of ion channels and osmotic imbalance of body fluids might be involved in the occurrence of pericardial and yolk sac edema in *ptch1* mutants (Figure S7E).

Based on these observations, we speculate that the heart insufficiency and defects in the formation of the vascular network in *ptch1* mutants might cause blood leakage and coagulation, and that the heart ischemia and defects in atrial ventricular differentiation might cause myocardial injury and an elongated atrium, which might contribute to pericardial edema. However, further research and more data are needed to confirm these hypotheses.

Angiogenesis is regulated by multiple pathway such as VEGF, Notch, JAK/STAT, and PI3K pathways [25,47,48]. *Vegf* is a marker for arterialization and is upregulated by activation of the Hh pathway [49,50]. In zebrafish, double mutations of *ptch1* and *ptch2* convert the posterior cardinal vein into a second artery through the regulatory processes of downstream VEGF and/or Notch signaling pathways [30]. In our study, *vegfa* was significantly upregulated in the *ptch1* mutants (Figure 6D). Lumen dilatation in the aortic arch and the failure of venous network formation in the yolk sac were consistently observed (Figure 5E–H), which suggests the possibility of excessive arterialization. On the other hand, *Vegf* is also one of the angiogenesis-promoting genes along with *Junb*, *Eef2k*, and *Angptl4*, and some studies have reported the role of these genes in pathological conditions. *Vegf* can be targeted and transcriptionally activated by HIF-1 in a hypoxic microenvironment to promote angiogenesis of hepatocellular carcinoma and melanoma tumors [51,52,53]; *Eef2k* can promote angiogenesis of liver cancer cells through PI3K/Akt and Stat3 [54]; and *Angpt4* can promote the proliferation, survival, and invasion of tumor cells, as well as the expansion of vascular tumors [55]. In addition, Socs3 (a negative feedback regulator) plays a role in regulating the JAK/STAT pathway, and it is also a key endogenous feedback inhibitor of pathologic angiogenesis [56], which can inhibit the proliferation and angiogenesis of cancer cells by downregulating the activation of Akt but not Stat3 [57]. In our study, not only the genes related to angiogenesis promotion, including *vegfa*, *junb*, *eef2k*, and *angptl4*, but also *socs3* were significantly upregulated. Furthermore, the upregulated expression levels of these genes in the *ptch1* mutants can be restored to that in WT through the antagonist of Smo treatment. Therefore, our study further confirms that multiple genes including *socs3*, *vegfa*, *junb*, *eef2k*, and *angptl4* are involved in the regulation of angiogenesis via the Hh pathway.

Hh signaling can be transmitted through either the Hh canonical pathway or the Smo-independent and/or Gli-independent Hh noncanonical pathway [58]. Cyclopamine (an antagonist of Smo) has been reported to block the transduction of Hh canonical signaling in a dose-dependent manner by inhibiting Smo, and it is a drug used clinically to treat Hh pathway-related diseases and cancers [59,60]. In preliminary experiments, we found that treatment with 10 μM cyclopamine inhibited transduction of Hh signaling, while it did not cause the developmental dysplasia affected by drug toxicity in Nile tilapia early larvae. Furthermore, in this study, by treating the *ptch1* mutants with 10 μM cyclopamine, the mRNA expression levels of Hh downstream members (*smo* and *gli2* but not *gli1*) were rescued to the level of WT (Figure 6D), and the abnormal phenotypes in the *ptch1* mutants were also greatly rescued (Figure S8). These results suggest that Ptch1 might function in angiogenesis and maintenance of vascular integrity through a Smo-dependent and Gli-dependent canonical pathway. However, we cannot rule out the possibility that Ptch1 might also function through a Smo-independent noncanonical pathway. For example, cyclopamine treatment cannot completely rescue *ptch1* mutant phenotypes, especially those with edema (Figure S8). Nonetheless, it is a unfortunate that we failed to obtain *ptch1* homozygous mutant progeny because of its lethal effect at the early larval stage, which was supported by the fact that all of the sperm produced by F0 mutants had no frameshift mutations but only non-frameshift mutations (Table S2 and Figure S9).

## 4. Materials and Methods

### 4.1. Animals

Nile tilapia were raised in a circulating, aerated freshwater tank under natural light at 26 ± 0.5 °C. All progeny were obtained by crossing normal XY males with normal XX females. All animal experiments were carried out in accordance with the regulations of the Guide for Care and Use of Laboratory Animals and approved by the Institutional Animal Care and Use Committee of Southwest University (NO. IACUC-20181015-12).

### 4.2. Sample Collection, RNA Extraction, and cDNA Synthesis

Nile tilapia tissues including brain, gill, heart, intestine, kidney, liver, muscle, ovary, spleen, and testis, and embryos at 1 hpf (hour post fertilization), 2 hpf, 4 hpf, 10 hpf, 15 hpf, 20 hpf, 45 hpf, 65 hpf, and 75 hpf were collected. Total RNAs were extracted according to the instructions of the RNAiso PLUS reagent (Takara Bio Inc., Otsu, Japan), treated with RNase-free DNase I (Thermo Scientific Corp, Waltham, MA, USA), and then reversely transcribed to cDNAs using the PrimeScript™ RT reagent Kit (Takara Bio Inc., Otsu, Japan).

### 4.3. Cloning and Sequence Analysis

The putative coding sequence containing the full open reading frame (ORF) of Nile tilapia *ptch1* was obtained by searching GenBank (GenBank accession no. XM_013271894.3) and bioinformatics analyses. PCR primers specific for *ptch1* were designed (Table S1). PCR was run for 35 cycles of 30 s at 95 °C, 60 °C for 30 s, and 72 °C for 30 s, followed by further extension at 72 °C for 10 min. PCR products were resolved by agarose gel electrophoresis. Bands of the expected size were purified, subcloned, sequenced, and further analyzed as previously described [61]. Briefly, the BLAST program (https://blast.ncbi.nlm.nih.gov/Blast.cgi, 10 January 2023) was used to search gene sequences from other species in the NCBI (http://www.ncbi.nlm.nih.gov, 10 January 2023); the ClustalX 1.83 program and GeneDoc 2.6 software were used to analyze multiple amino acid sequence alignment and identity; MEGA 7.0 software was used to construct neighbor-joining phylogenic trees; and the Ensembl Genome Browser (http://www.ensembl.org, 10 January 2023) was used for gene syntenic analyses.

### 4.4. Reverse Transcription PCR (RT-PCR) Analysis

The mRNA expression of Nile tilapia *ptch1* in different embryonic development stages (1 hpf, 2 hpf, 4 hpf, 10 hpf, 15 hpf, 20 hpf, 45 hpf, 65 hpf, and 75 hpf) and adult tissues was detected by RT-PCR. Primers specific for *ptch1* with a spanning intron were designed (Table S1), and a single band was detected by agarose electrophoresis. PCR was run for 32 cycles of 30 s at 95 °C, 57 °C for 30 s, and 72 °C for 30 s, followed by further extension at 72 °C for 10 min. Simultaneously, *β-actin* was amplified as an internal control. Meanwhile, the mRNA expression levels of selected genes in *ptch1* mutant and WT early larvae were evaluated by quantitative RT-PCR (RT-qPCR) using the SYBR1 Premix Ex TaqTM II kit (Takara, Tokyo, Japan) and the ABI-7500 real-time PCR system (Applied Biosystems, Weiterstadt, Germany). Relative mRNA expression level was determined using the formula R = 2^−ΔΔCt^. The primers used for RT-qPCR are listed in Table S1.

### 4.5. In Situ Hybridization (ISH)

The ORF of Nile tilapia *ptch1* was amplified, and the amplicons were cloned into pGEM-T Easy Vector (Promega, Madison, WI, USA). Sense and anti-sense RNA probes were synthesized and were labeled with DIG by in vitro transcription using SP6 and T7 RNA polymerase (Promega, Madison, WI, USA) and a DIG RNA labeling kit (Roche, Mannheim, Germany).

The larvae at 6 dpf and gonads at the indicated times were fixed in 4% paraformaldehyde in phosphate-buffered saline (PBS) and processed for serial paraffin sectioning at 5 μm thickness. The sections were incubated at 65 °C for 16 h with the sense and antisense probes, respectively, then incubated with Anti-DIG-POD (1:3000, 1% blocker dilution) for 3 h at room temperature. Chromogenic and fluorescent ISH staining was done with BCIP/NBT substrates (Roche) and TSA Plus Fluorescence Systems (NEN Life Science, Boston, MA, USA), respectively. Nuclear staining was done by DAPI in the Gold Antifade reagent (Invitrogen, Waltham, MA, USA). Fluorescence signals were captured by confocal microscopy (Olympus FV3000) (Olympus, Tokyo, Japan).

### 4.6. ptch1 Mutations by CRISPR/Cas9

The gRNA target (GGAGGCGCTCCTGCAGCACCTGG) located on exon 3 of Nile tilapia *ptch1* was designed (https://crispr.dbcls.jp, 6 February 2023) and synthesized. *ptch1* gRNA and Cas9 mRNAs were prepared using the Mega Script T7 Kit (Ambion) as previously described [62]. For the *ptch1* mutant group, embryonic animal pole was injected with the combinations of *ptch1* gRNA (500 ng/μL) and Cas9 mRNAs (1000 ng/μL) at the 1-cell stage of fertilized embryos. For the control group, the control embryos were injected with the same concentration of *ptch1* gRNA (500 ng/μL) or Cas9 mRNAs (1000 ng/μL) alone to exclude the influence of external RNAs on embryonic development. For the detection of *ptch1* mutation rate, samples of *ptch1* mutant group embryos (2 dpf) and larvae (6 dpf) were collected, and genomes were extracted by the phenol chloroform–isoamyl alcohol extraction method. The target fragments of *ptch1* were amplified by specific primers (Table S1) and separated by polyacrylamide gel electrophoresis (PAGE). The gray values of the band at 150 bp (original band) and outside 150 bp (mutant bands) were quantified using Image J (version 1.54i) software. The *ptch1* mutation rate of each embryo was calculated by dividing the gray value of the mutant bands to that of the total bands, which include the original band and mutant bands.

### 4.7. Classification of Mutants and Calculation of Area

Embryonic and larval development at different stages (1–6 dpf) was observed and evaluated using a stereomicroscope (Leica DFC310 FX, Wetzlar, Germany). For the normal development stage of Nile tilapia, the immature heart (i.e., heart tube) and the initiation of blood circulation can be seen at 4 dpf, and the differentiation of the heart and the branching of more vascular network can be seen at 5–6 dpf [34]. For the *ptch1* mutant group, the mutants were divided into two categories, i.e., *ptch1* mutant Ⅰ and *ptch1* mutant Ⅱ, based on the severity of the phenotype and the stage of occurrence. The *ptch1* mutant I was defined as a mutant that occurred at the early stage of 4 dpf, without a yolk sac vascular network, no apparent bloodstreams, severe pericardial edema, and a highly stretched linear heart at 5 dpf, and could not survive at 4–5 dpf. The *ptch1* mutant Ⅱ was defined as a mutant that occurred at the late stage of 5 dpf, characterized by circulatory defects, blood leakage and coagulation in multiple tissues, pronounced pericardial edema, and a moderately stretched heart, and was not viable at 6–8 dpf.

For area measurement of the of specific tissues, each larva was photographed under the same conditions (the view and magnification of the stereomicroscope), and the area was calculated using Image J (version 1.54i) software after the calibration of the scale bar.

### 4.8. Hematoxylin and Eosin (H&E) Staining

The early larvae (5–6 dpf) were fixed in Bouin’s solution for 24 h at room temperature. The fixed samples were then processed as follows: serial dehydration in 70%, 80%, and 90% ethanol for 1.5 h each, 95% ethanol for 2 h, and 100% ethanol three times for 1 h each; sequential clearance in xylene and ethanol mixture (1:1) for 30 min and xylene twice for 30 min each; and infiltration in paraffin for 2 h. The samples were sectioned at 5 µm thickness (Leica Microsystems, Wetzlar, Germany) and then stained with H&E. Images were captured with an Olympus BX51 light microscope (Olympus, Tokyo, Japan).

### 4.9. RNA Sequencing (RNA-Seq)

Five early larvae from the *ptch1* mutants or WT were sampled at 5 dpf, respectively. Total RNAs from the mutant group or the WT group were extracted by the method mentioned above. The transcripts were sequenced using the BGI-SEQ sequencing platform (BGI, Wuhan, China). All clean reads were mapped to the Nile tilapia genome sequence (https://ftp.ncbi.nlm.nih.gov/genomes/all/GCF/001/858/045/GCF_001858045.2_O_niloticus_UMD_NMBU/, 18 April 2023). Gene expression was quantified using RSEM (version 1.2.8) software and the method of fragments per kilobase of exon model per million mapped fragments (FPKM). Differentially expressed genes (DEGs) were analyzed among different samples using the DESeq2 with Q value ≤ 0.05. Furthermore, Gene Ontology (GO) and Kyoto Encyclopedia of Genes and Genomes (KEGG) enrichment analysis of annotated different-expression gene was performed by Phyper in R software (version 4.2.3) based on a hypergeometric test. Significant levels of terms and pathways were corrected by the Q value with a rigorous threshold (Q value ≤ 0.05).

### 4.10. Drug Treatment

To investigate whether Ptch1 mediates cardiovascular development through Smo signaling, 10 μM cyclopamine (MCE, Monmouth Junction, NJ, USA), an Smo antagonist, was used to treat some of the *ptch1* mutants. At the same time, the same concentration of cyclopamine or DMSO was used to treat the WT embryos to evaluate the toxicity of the drug itself. All embryos were injected at the 1-cell stage as described above, and larvae were observed and sampled at 6 dpf.

### 4.11. Data Analysis

Statistics analyses were performed using GraphPad Prism 8 software package (GraphPad Software, La Jolla, CA, USA), data are showed as mean ± SD from at least three independent experiments in triplicates, and statistical significance was analyzed by single-factor analysis of variance and two-tailed Student’s *t*-test (confidence interval 95%). Statistical significance is indicated as follows: * *p* < 0.05; ** *p* < 0.01; *** *p* < 0.001; NS, not significant.

## 5. Conclusions

In summary, using Nile tilapia as a model, our data comprehensively demonstrate that Ptch1 is important for cardiovascular development and vascular integrity. *ptch1* mutations lead to abnormal heart development and angiogenesis defects, blood leakage and coagulation, and ultimately death of early larvae. However, these can be greatly rescued through Smo antagonist treatment. These findings indicate that Nile tilapia *ptch1* deficiency can disrupt the cardiovascular development and vascular integrity through Smo signaling. Our study enriches and deepens our understanding of Hh signaling in cardiovascular biology, and provides an excellent animal model to promote the development of the therapeutic potential of Hh signaling pathway components for cardiovascular diseases.

## Figures and Tables

**Table 1:** Amino acid sequence identity of Ptch1 among different species (%).

Species	Full Length	Domain						
		N^cyto^	Loop1	SSD1	ML^cyto^	Loop2	SSD2	C^cyto^
*Oreochromis niloticus*	100	100	100	100	100	100	100	100
*Danio rerio*	76	86	81	99	55	85	97	53
*Homo sapiens*	69	64	80	98	63	79	93	43

Loop, extracellular loop domain; SSD, sterol-sensing domain; N^cyto^, cytoplasmic domain of N-terminal; ML^cyto^, cytoplasmic domain of middle loop; C^cyto^, cytoplasmic domain of C-terminal.

**Table 2:** Number and proportion of larvae with related phenotypes at 6 dpf.

Group	Total Larvae Number	Number of Larvae with the Phenotype			Proportion of Larvae with High Mutation Rate (Phenotype of E+)	Proportion of Viable Larvae (Phenotype of C−)
		C−, E−	C+, E+	C−, E+		
WT	336	336	0	0	-	100%
10 μM Cyc	239	239	0	0	-	100%
*ptch1* gRNA	232	95	137	0	59.05%	40.95%
*ptch1* gRNA +10 μM Cyc	268	126	78	64	54.10%	70.90%

Cyc, cyclopamine. Statistics were obtained from three sets of biological replicates.

## Data Availability

The datasets used and analyzed during the current study are available from the corresponding author on reasonable request.

## Supplementary Materials

The following supporting information can be downloaded at: https://www.mdpi.com/article/10.3390/ijms25063321/s1.
