# Supplementary material for: Nile Tilapia (Oreochromis niloticus) Patched1 Mutations Disrupt Cardiovascular Development and Vascular Integrity through Smoothened Signaling
Source: Int J Mol Sci. 2024 Mar 15;25(6):3321. doi: 10.3390/ijms25063321 (PMC10970307; doi:10.3390/ijms25063321)
Supplement: Supplementary file 1 [file ijms-25-03321-s001.zip › Figure caption.pdf]

The following text is the legend and caption for supplementary figures.

Supplementary Figures

Figure S1. Multiple sequence alignments of Ptch1 amino acids in Nile tilapia, zebrafish and human. Loop, extracellular loop domain; SSD, sterol sensing domains; N^{cyto}, cytoplasmic domain of N-terminal; ML^{cyto}, cytoplasmic domain of middle loop; C^{cyto}, cytoplasmic domain of C-terminal.

Figure S2. Analysis of Ptch1 mRNA expression patterns during embryonic development and early larval stages. (**A**) RT-PCR analysis was performed with β-actin serving as the internal reference. (**B**) Investigation of Ptch1 mRNA expression in early larvae (6 dpf) utilizing In Situ Hybridization (ISH). Larval paraffin sections underwent hybridization with *ptch1* antisense (**B1**) and control sense (**B2**) RNA probes. Black dashed lines indicate the heart (h), brain (b), and eye (e). Enlarged views of the heart (**B3**), brain (**B4**), and eye (**B5**) in **B1** are displayed. hpf, hours post fertilization. dpf, days post fertilization. Scale bars, **B1**, **B2**, 200 μm; **B3**, **B4**, **B5**, 50 μm.

Figure S3. The expression profile of *ptch1* mRNA in various tissues and its spatiotemporal expression pattern in gonads. (**A**) RT-PCR was utilized to determine the expression profile in adult tissues, with β-actin serving as the internal control. (**B**) The spatiotemporal expression pattern in gonads by ISH. Sections of testes and ovaries embedded in paraffin were hybridized with *ptch1* antisense and control sense RNA

probes, followed by development via fluorescence (red) subsequent to DAPI staining (blue). Images of gonadal sections pre-meiosis (testes at 60 days after hatching (dah) and ovaries at 30 dah) and post-sexual maturation (testes and ovaries at 180 dah) are presented. A dashed box shows the magnified image. Scale bars, 100 μm (for 30 dah and 60 dah); 50 μm (for 180 dah).

Figure S4. Comparison of WT and *ptch1* mutant embryos during early development. Dark field images of WT and *ptch1* mutant embryos were taken at 24-75 hpf, showing no apparent differences during early developmental stages. hpf, hours post fertilization. Scale bar, 500 μm.

Figure S5. Analysis of vascular distribution in various tissues of larvae at 4 dpf and 6 dpf. The numbers in **A1**-**A4** represent the proportion of larvae with this phenotype to the total number of larvae in the control or mutant group at the indicated stages, respectively. Compared with the WT larvae, the *ptch1* mutants showed impaired vascular establishment (*ptch1* mutant I) and blood leakage (*ptch1* mutant II) at 4 dpf and 6 dpf. Scale bars, **A1**-**B4**, 500 μm; **C1**-**C4**, 250 μm; **D1**-**D4**, 200 μm; **E1**-**F4**, 50 μm.

Figure S6. Abnormal eye morphology in *ptch1* mutants. (**A**) Abnormal morphology of the eye. The area of the eye (marked by black circle) and the lengths of the long and short axis (indicated by red dashed lines) of the eye were measured in WT and *ptch1* mutant larvae. (**B**) Quantification of eye. The eye areas of both WT and *ptch1* mutant larvae were quantified using Image J (version 1.54i) software under identical magnification (n = 15). (**C**) Analysis of eye aspect ratio. The eye aspect ratio was

calculated by dividing the short axis diameter by the long axis diameter. A ratio close to 1.0 indicates a round shape typical of WT larvae. Significant differences versus the control are indicated by ***$p < 0.001$. Scale bar, 200 μm.

Figure S7. Analyses of DEGs of *ptch1* mutants. (**A**) KEGG enrichment analysis of down-regulated DEGs in *ptch1* mutants. (**B**) KEGG enrichment analysis of up-regulated DEGs in *ptch1* mutants. (**C**) KEGG enrichment analysis of DEGs related to circulatory system. (**D**) GO analysis of DEGs related to the circulatory system. Generally, a function with a Q value < 0.05 is considered to be significantly enriched according to statistical analysis. (**E**) Expression levels of the selected genes related to ion channel protein following *ptch1* mutation. FPKM, Fragments Per Kilobase of exon model per Million mapped fragments.

Figure S8. Cyclopamine treatment partially rescued blood circulation deficiencies in *ptch1* mutants. Dark field and bright field images of cyclopamine treated larvae at 6 dpf are shown, respectively. The numbers in **D**-**F** represent the proportion of early larvae with this phenotype to the total number of early larvae. The white circles highlight areas of blood leakage and coagulation (**B**,**E**), and the white arrows point to the swelling observed in the pericardial cavity and ventral vitelline areas (**B**,**C**). Coagulation−, Edema− (C−, E−): the larvae with normal blood circulation and pericardial cavity, and no edema of pericardial and dorsal vitelline. C−, E+: the larvae with normal blood circulation and pericardial cavity, but had dorsal vitelline edema. C+, E+: the larvae with blood leakage and coagulation, pericardial edema and dorsal vitelline edema. IsV, intersegmental blood vessel; DA, dorsal aorta; PCV, profundal caudal vein; N,

notochord. The statistics were derived from three sets of biological replications. Scale bars, **A-F**, 500 μm; **G-I**, 200 μm.

Figure S9. A sequence illustrating a non-frameshift mutation found in the haploid sperm of *ptch1* mutants.
