# Supplementary figures and images for: Nile Tilapia (Oreochromis niloticus) Patched1 Mutations Disrupt Cardiovascular Development and Vascular Integrity through Smoothened Signaling
Source: Int J Mol Sci. 2024 Mar 15;25(6):3321. doi: 10.3390/ijms25063321 (PMC10970307; doi:10.3390/ijms25063321)

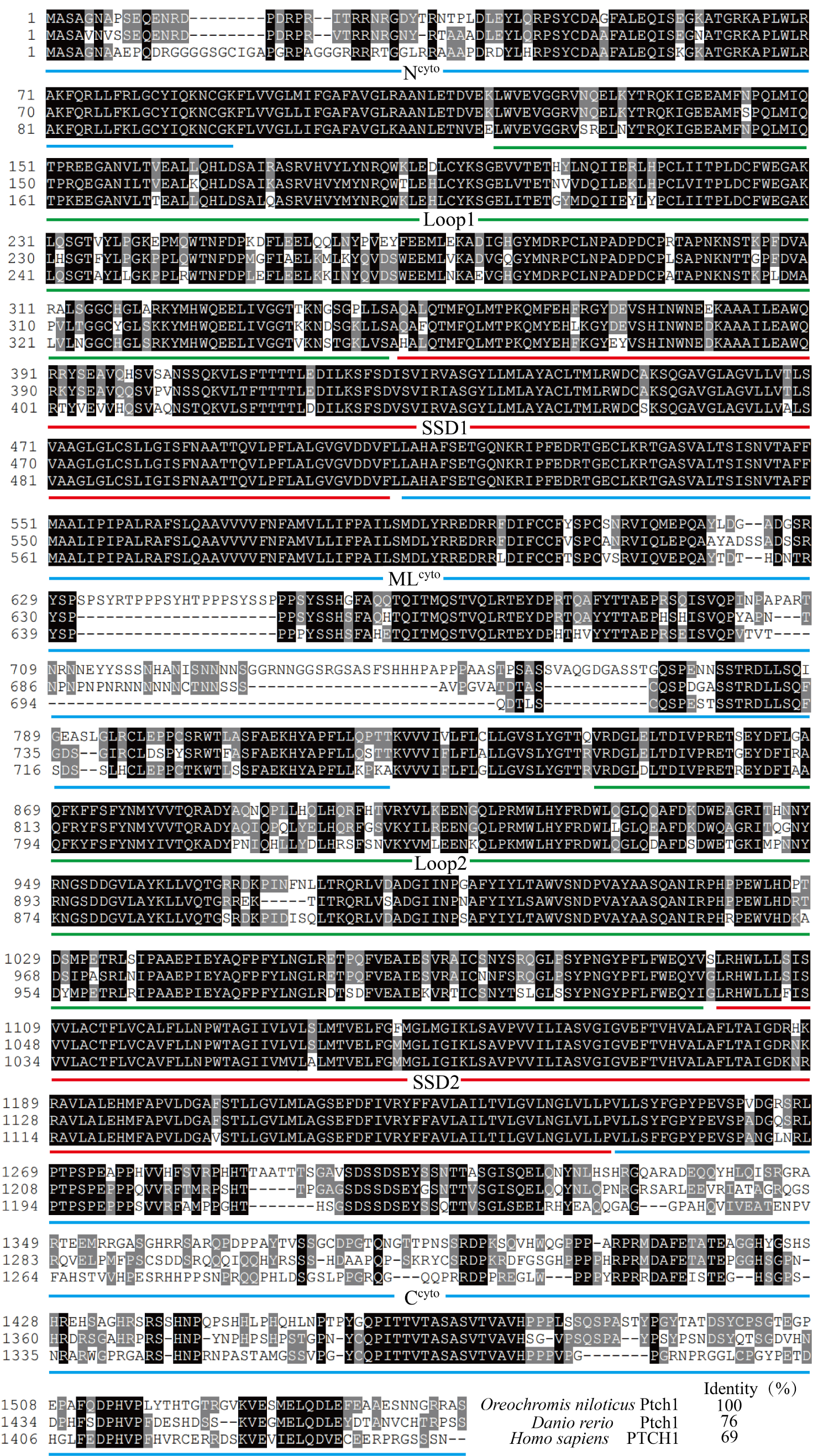

Supplement: Supplementary file 1 [file ijms-25-03321-s001.zip › Supplementary Figures/Figure S1.tif]

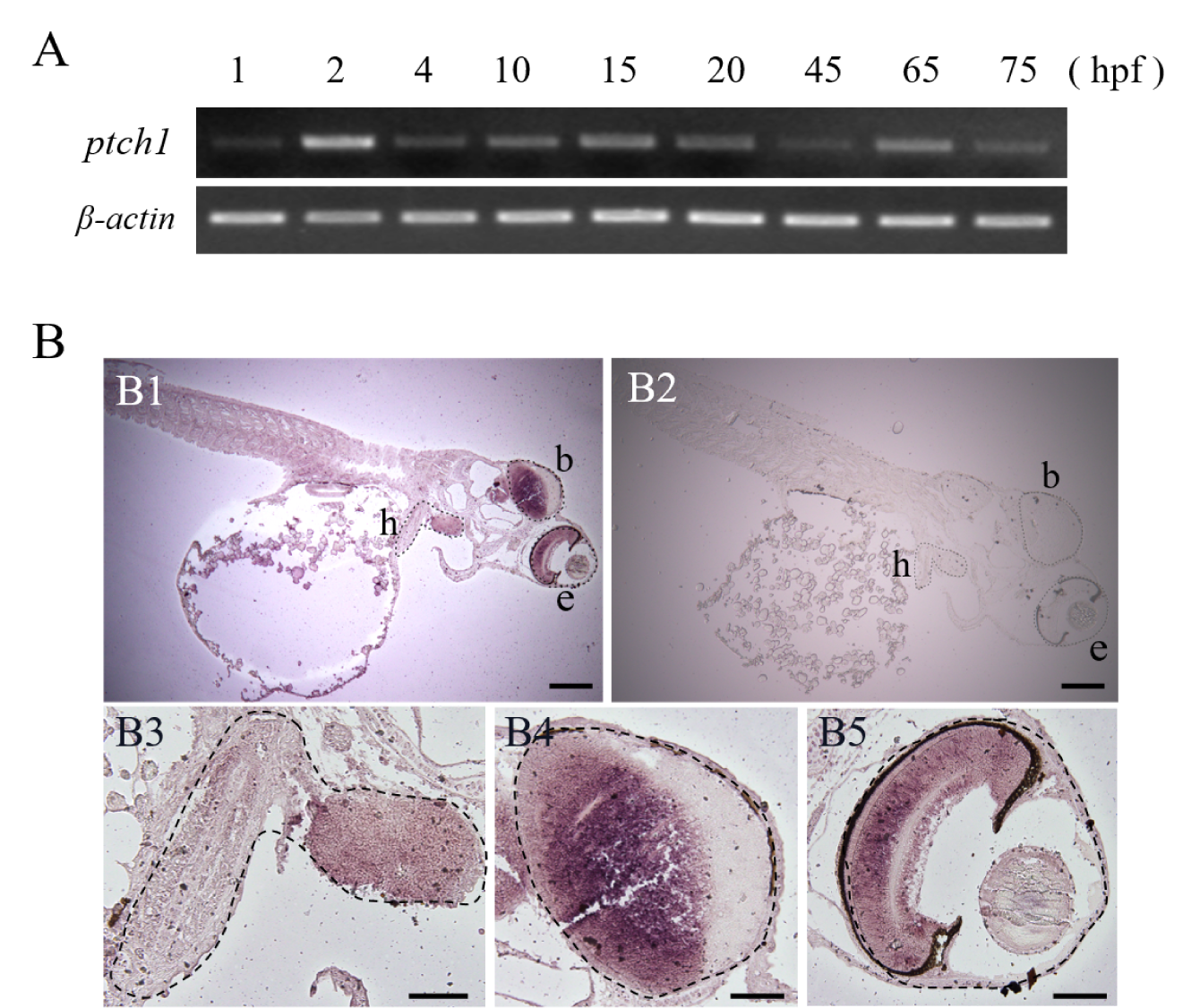

Supplement: Supplementary file 1 [file ijms-25-03321-s001.zip › Supplementary Figures/Figure S2(Revised).tif]

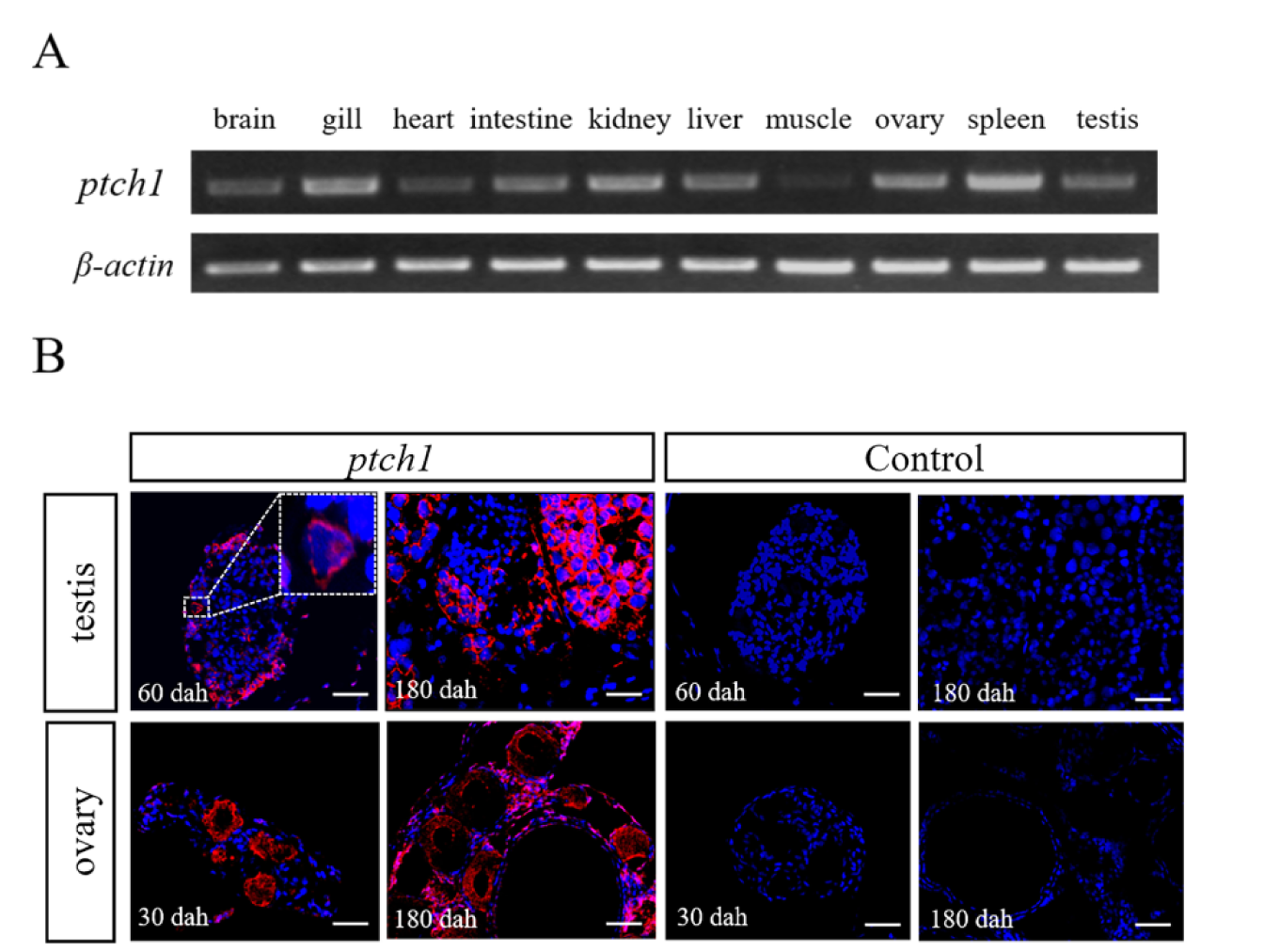

Supplement: Supplementary file 1 [file ijms-25-03321-s001.zip › Supplementary Figures/Figure S3.tif]

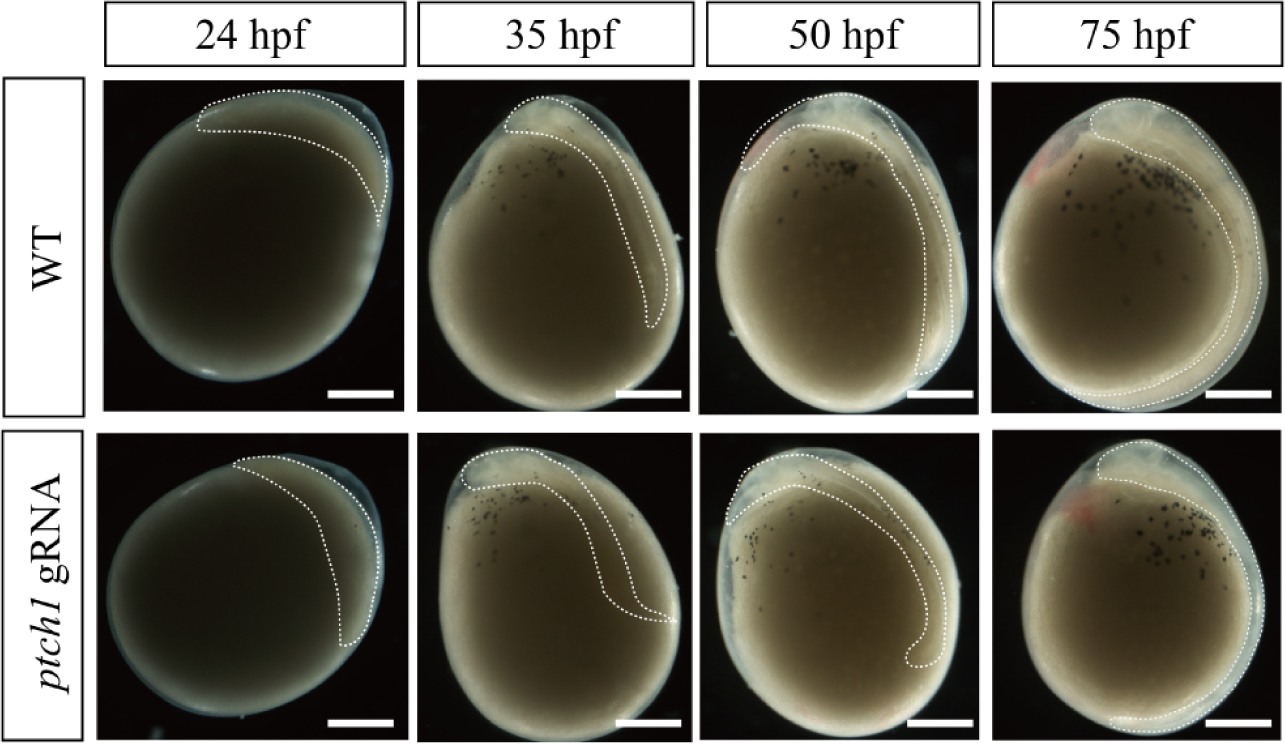

Supplement: Supplementary file 1 [file ijms-25-03321-s001.zip › Supplementary Figures/Figure S4.tif]

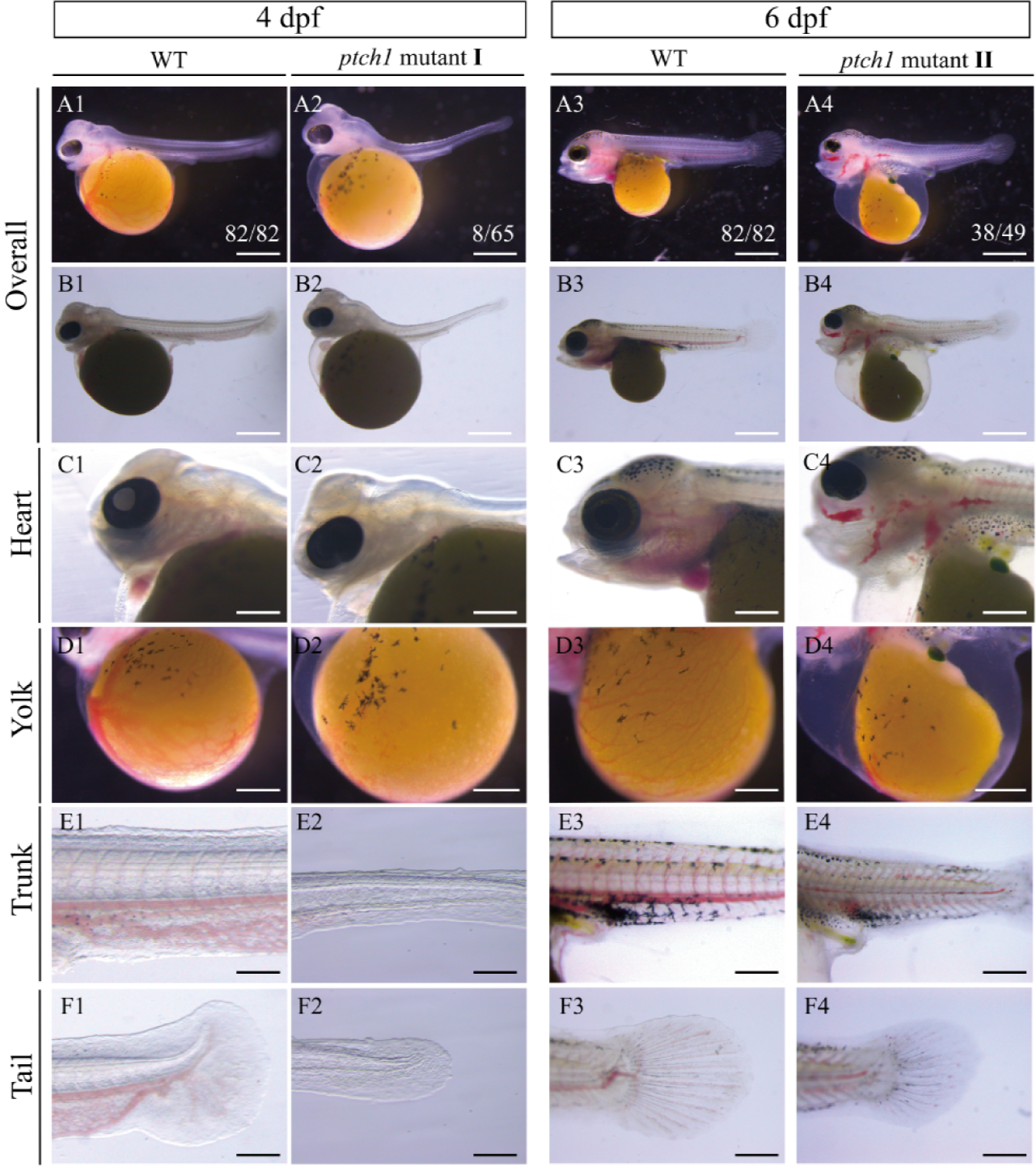

Supplement: Supplementary file 1 [file ijms-25-03321-s001.zip › Supplementary Figures/Figure S5.tif]

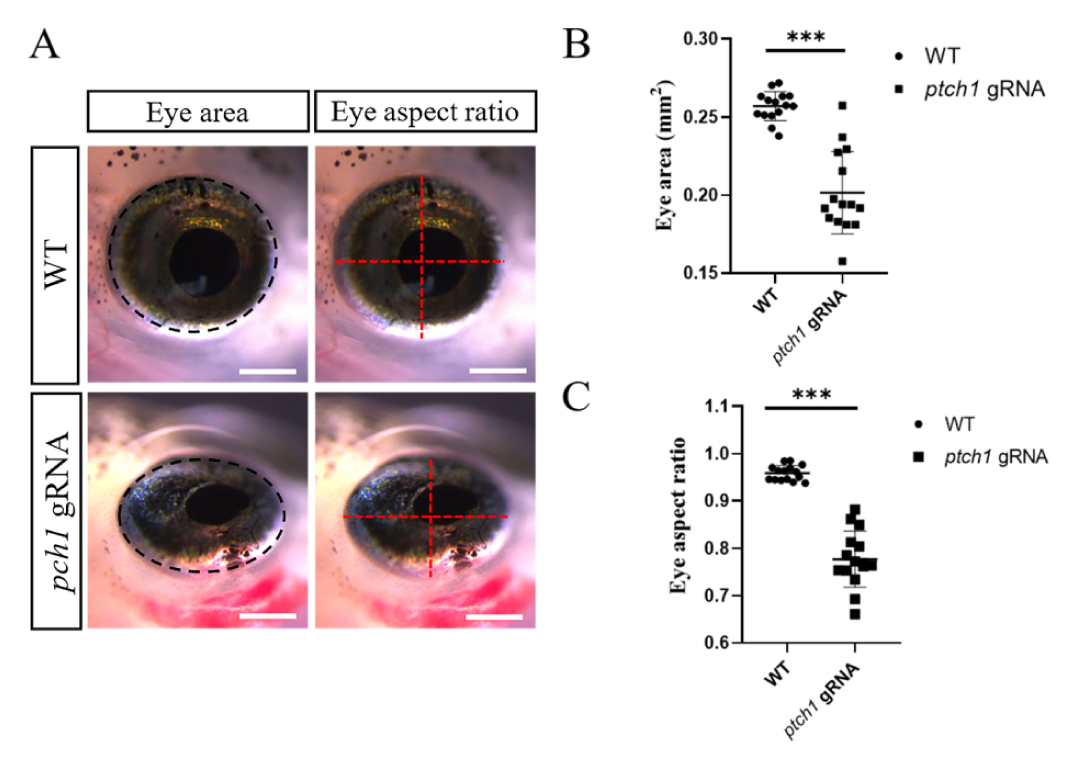

Supplement: Supplementary file 1 [file ijms-25-03321-s001.zip › Supplementary Figures/Figure S6.tif]

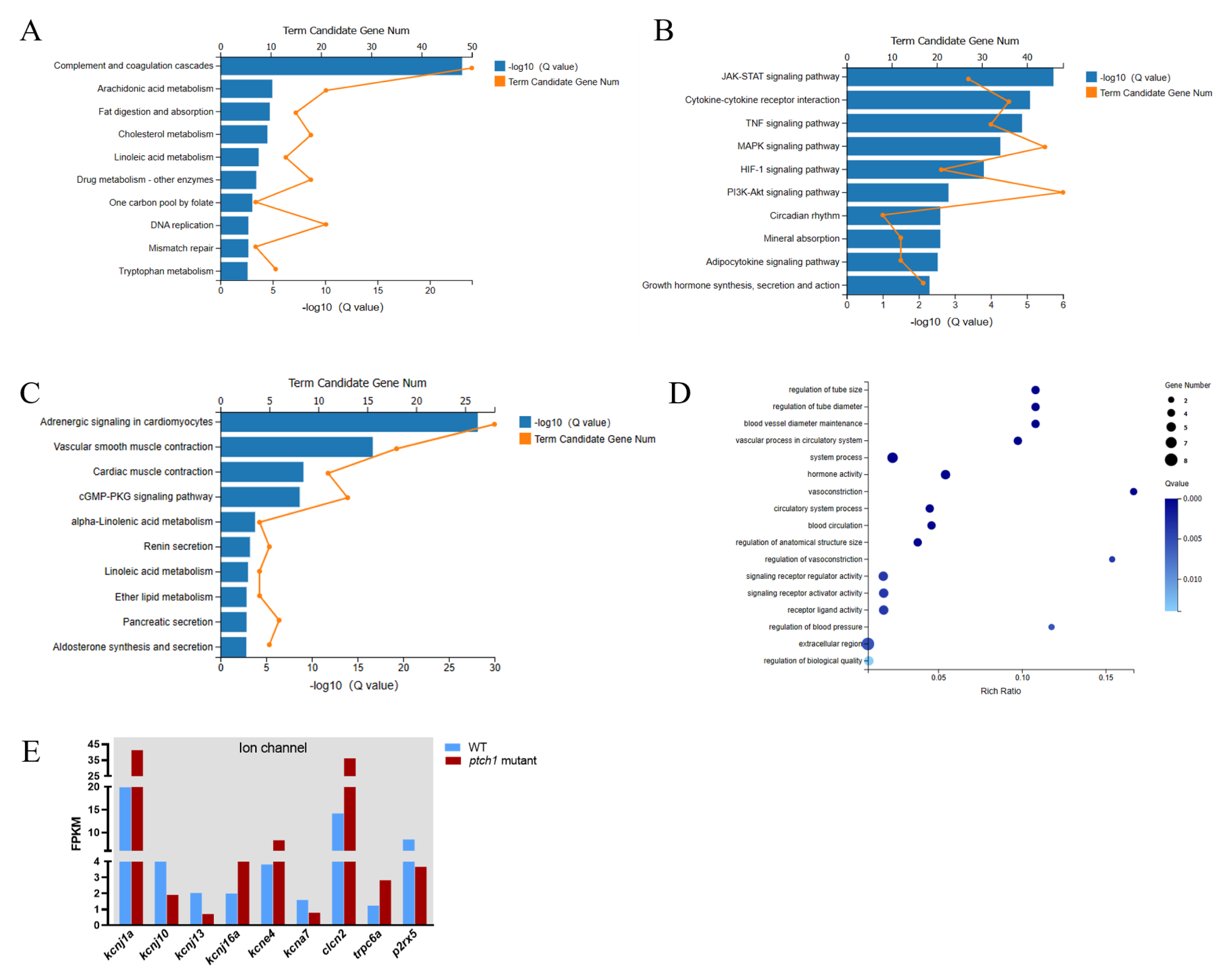

Supplement: Supplementary file 1 [file ijms-25-03321-s001.zip › Supplementary Figures/Figure S7.tif]

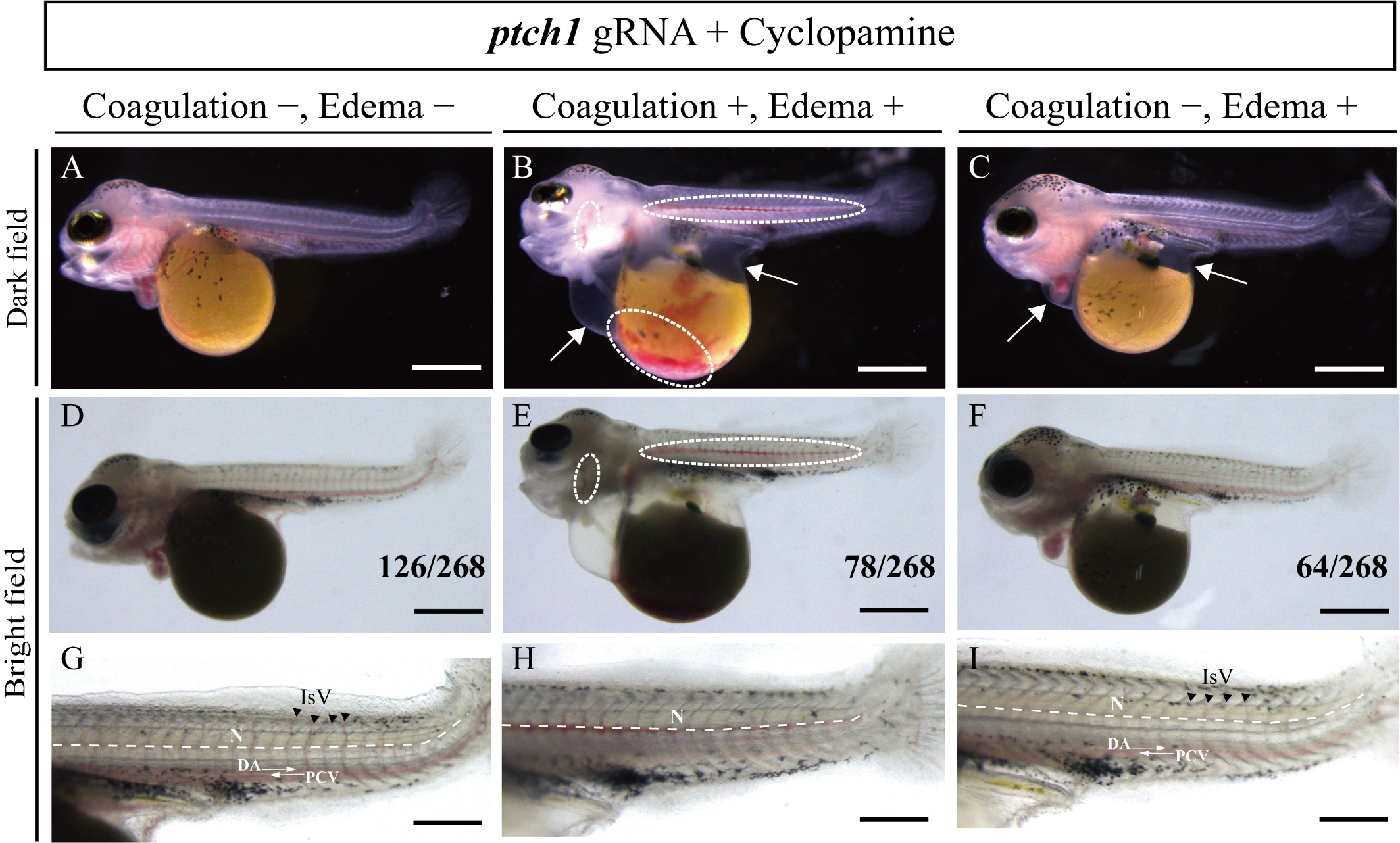

Supplement: Supplementary file 1 [file ijms-25-03321-s001.zip › Supplementary Figures/Figure S8(Revised).tif]

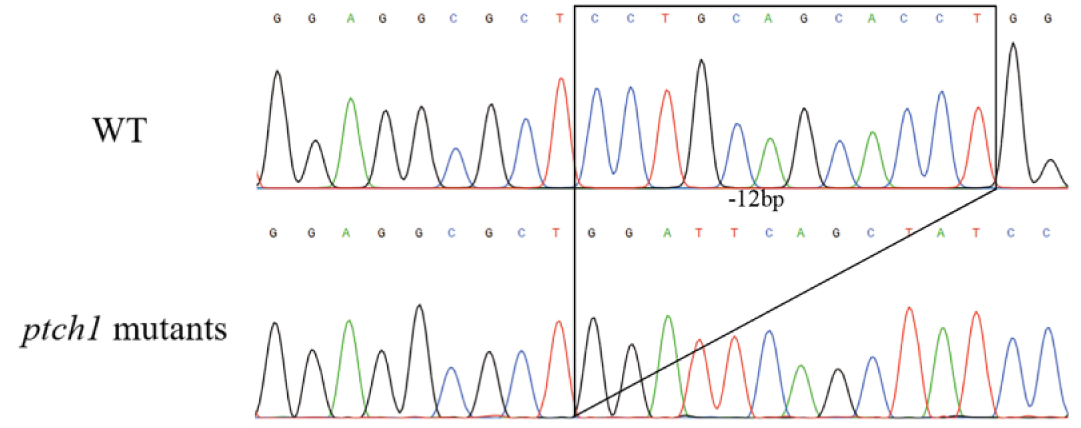

Supplement: Supplementary file 1 [file ijms-25-03321-s001.zip › Supplementary Figures/Figure S9.tif]
